# Angiographic patterns of coronary artery disease in young patients presenting at a tertiary cardiac center

**DOI:** 10.12669/pjms.38.8.6162

**Published:** 2022

**Authors:** Abdul Wajid Khan Faisal, Ghalib Habib, Samra Yasmin, Waqas Latif, Sheeraz Ahmed

**Affiliations:** 1 Abdul Wajid Khan Faisal, FCPS (Medicine); FCPS (Cardiology). Professor of Cardiology, Department of Cardiology, Punjab Institute of Cardiology, Lahore, Pakistan; 2 Ghalib Habib, FCPS (Cardiology). Senior Registrar Cardiology, Department of Cardiology, Punjab Institute of Cardiology, Lahore, Pakistan; 3 Samra Yasmin, FCPS (Medicine); FCPS (Cardiology) Assistant Professor of Cardiology, Department of Cardiology, Punjab Institute of Cardiology, Lahore, Pakistan; 4 Waqas Latif, MSc. Biostatistics (PU), M. Phil. Statistics (UOS) Biostatistician, University of Health Sciences, Lahore, Pakistan; 5 Sheeraz Ahmed, BCS. IT Professional, Punjab Institute of Cardiology, Lahore, Pakistan

**Keywords:** Angiographic patterns, Coronary artery disease, Young age

## Abstract

**Objective:**

To compare the patterns of coronary artery disease (CAD) between young adults ≤35 years of age and patients >35 years of age.

**Methods:**

The observational retrospective study was carried out in angiography department of emergency at Punjab Institute of Cardiology, Lahore between January 2020 and October 2020. Patients ≤35 years old were in Group-I whereas patients >35 years who served as controls were in Group-II. Patients with unstable angina, non-ST and ST elevation MI all were included. The patients with previous history of CAD (CABG/PCI) and angiography done for other purposes i.e., before valvular surgery or PPM implantation were excluded.

**Results:**

Out of 1268 patients, 552 were in Group-I and 716 were in Group-II. The prevalence of normal coronaries/ mild CAD was higher in Group-I i.e., 224(40.6%) than in Group-II i.e., 64 (8.9%). Single vessel disease (SVD) was comparable in both the groups 185 (33.5%) vs. 216 (30.2%). Double vessel disease and triple vessel disease (TVD) was common in Group-II and left main stem (LMS) involvement was also significantly higher in Group-II i.e., 32 (4.5%) vs. 8 (1.4%). Clot in coronary arteries with or without underlying CAD was seen more frequently in Group-I, 61(11.1%) vs. 34 (4.7%). Presence of clot was seen mostly in those patients who had moderate coronary artery disease.

**Conclusion:**

Young patients have different coronary artery disease patterns, so the management strategy must be different in this population. Majority of the young patients have non severe disease. Clot formation is commonly seen in young adults with moderate CAD.

## INTRODUCTION

Worldwide, coronary artery disease (CAD) ranks as one of the leading causes of death for both genders^1^. The age of CAD is declining in developing nations, which is related to economic and social factors^2^. As the number of risk factors rises, the rate of CAD^3^ increases accordingly. Risk factors, angiographic results, and clinical circumstances are varied in young and old patients, and these differences have an impact on prognosis^4^. If the right investigations and management are carried out, a better prognosis for CAD in young adults can be attained.^5^ Difference in gender is also important regarding clinical manifestations, diagnosis, management and prevention of CAD.^6^

Angiographically the young patients are different from older patients, in that they have normal coronary arteries or single vessel disease as compared to complex disease in older patients, so the management required in these patients may be different^7^. Smoking, high blood pressure, and family history are the three main risk factors for CAD in young adults as studied by Zeitouni et al^8^. The pattern of CAD is important to know because it affects the treatment options and disease outcome. This study was conducted to compare the pattern of CAD in young (<35 years) and patients >35 years of age.

In another study from Pakistan conducted by Khan HU et al.,^9^ showed that normal vessels were common in young patient (<40 years of age) also had less severe CAD and left main stem involvement was extremely rare. Acute coronary syndrome (ACS) is uncommon in young patients under 40 years of age and these young patients have a different CAD pattern in comparison with older patients.

## METHODS

This was an observational retrospective study. We studied angiographic patterns of young patients (≤35 years) presenting in Emergency department at Punjab Institute of Cardiology between January 2020 and October 2020 after the approval from local ethical review board (Ref.No: RTPGME-Research-145 dated November 26, 2020). There were two groups, i.e., Group-I and Group-II. Cases in Group-I were patients ≤35 years of age, while controls in Group-II were patients >35 years of age. The study comprised patients of either gender with unstable angina, non-ST elevation MI, or ST elevation MI. Coronary angiography was performed of all patients during the index hospitalization. Patients having a history of coronary artery disease (CABG/PCI ), angiography performed for other reasons, such as before pacemaker implantation, congenital heart disease, and valvular heart disease surgery were excluded. Five hundred fifty two patients were enrolled in Group-I after meeting the inclusion and exclusion criteria. Three thousand five hundred patients in Group II met the criteria, and 716 of them were randomly selected.

Significant CAD was defined as ≥50% narrowing of the diameter of the lumen of the coronary artery. Less than 50% stenosis was considered mild coronary artery disease, 50-70% stenosis was considered moderate, and more than 70% stenosis was considered severe. Chi Square test was applied using SPSS 23.0 software for data analysis, p-value <0.05 was considered as significant.

## RESULTS

We studied a total of 1268 patients. 552 patients were < 35 years of age (Group-I), minimum age was 16 years and maximum age was 35 years (31.40 ± 3.79 years mean age). Seven hunded sixteen patients were more than 35 years of age (Group-II) minimum age was 36 years and maximum age was 85 years (52.98 ± 8.65 mean age). 463(83.9%) male patients were in Group-I, while 564 (78.8%) male patients were in Group-II (Table-I).

**Table-I:** Baseline characteristics of study population

	Group-I ≤35 years	Group-II >35 years	Total Patients
Number of patients	552	716	1268
Age (Mean) years	31.40 ± 3.79	52.98 ± 8.65	
Age Range (years)	16-35	36-85	
Male	463 (83.9%)	564 (78.8%)	1027 (81%)
Female	89 (16.1%)	152 (21.2%)	241 (19%)

When we analyzed the coronary artery disease in both groups, it was found that mild coronary artery disease (CAD)/normal coronaries were more common in the younger age Group-I 224(40.6%) vs. 64 (8.9%) in Group-II p-value of 0.001. Single vessel disease (SVD) was comparable in both the groups 185 (33.5%) vs. 216 (30.2%). Double vessel disease (DVD) was significantly common in Group-II that is 184 (25.7%) vs. 74 (13.4%) p value<0.001. Triple vessel disease (TVD) and left main stem (LMS) involvement was also significantly higher in >35 years age group 252(35.2%) vs. 49(8.9%) p-value <0.001 and 32 (4.5%) vs 8 (1.4%) p value 0.002 respectively. Clot in coronary arteries with or without underlying CAD was seen more frequently in Group-I, 61(11.1%) vs. 34 (4.7%) p-value < 0.001 (Table-II).

**Table-II:** Angiographic patterns of patients in both groups

	Group-I (≤ 35 years)	Group-II (>35 years)	Total	P-value
Normal Coronaries / mild CAD	224 (40.6%)	64 (8.9%)	288 (22.7%)	0.001
Single vessel disease (SVD)	185 (33.5%)	216 (30.2%)	401 (31.6%)	0.204
Double vessel disease (DVD)	74 (13.4%)	184 (25.7%)	258 (20.3%)	<0.001
Triple vessel disease (TVD)	49 (8.9%)	252 (35.2%)	301 (23.7%)	<0.001
LMS disease	8 (1.4%)	32 (4.5%)	40 (3.2%)	0.002
Clot with or without CAD	61 (11.1%)	34 (4.7%)	95 (7.5%)	<0.001

On sub analysis of angiographic findings of young patients, majority of patients had normal or mild CAD 224 (40.6%). Moderate CAD was found in only 64 (11.6%), while severe disease was present in 200 (36.2%) patients. Totally occluded arteries without any evidence of underlying coronary arteries were seen in 64 (11.05%) (Table-III).

**Table-III:** Severity of disease in young patients ≤ 35 years.

Normal / Mild	224 (40.6%)
Moderate	64 (11.6%)
Severe	200 (36.2%)
Only total occlusion	64 (11.6%)
Total number	552 (100%)

Presence of clot was seen mostly in those patients who had moderate coronary artery disease. Clot was significantly less common in patients with mild or severe disease (Table-IV). What is the causal relation of moderate disease with clot formation needs to be studied further.

**Table-IV:** Presence of clot in young patients ≤ 35 years.

Severity of disease	Clot Percentage	p-value
Normal / Mild	8 / 224 (3.6%)	<0.00001
Moderate	32 / 64 (50%)	
Severe(out of them 3 had total occlusion)	17 / 200 (8.5%)	
Only total occlusion(all other vessels normal)	4 /64 (6.25%)	
Total number	61/552 (11.05%)	

## DISCUSSION

CAD is an epidemic worldwide which is linked with significant morbidity and mortality. Acute myocardial infarction in young is more commonly associated with male sex, positive family history and less commonly seen in hypertensive and diabetic patients in Pakistani population as studied by Batra et al^10^. The patterns of coronary involvement is different in young patients as compared to old patients in both genders.^11^,^12^ In our study, male dominance was noted in both groups. By Mohammad et al. and Maroszyńska-Dmoch et al.^13^,^14^, the population of young patients with CAD is pre-dominantly male and the most important CAD risk factors were dyslipidemia, smoking, hypertension and obesity.

According to the results of our study, we observed that the pattern of involved vessels was different between young and older patients. Two and three vessels disease in older population and single vessel disease in younger population were common. In a study by Prajapati et al.^15^ it was assessed that single vessel disease was more frequent in younger patients and three vessel disease was more prominent in elderly patients. Mahjoob and Yang et al.^16^,^17^ also reported in their studies that young adults are characterized by a less extensive coronary disease. Single vessel disease was common in young adults.

Ali et al. demonstrated the pattern of coronary artery disease in their study on young individuals with ACS. Results were 70.7% patients having one vessel CAD, 28.0% two vessel CAD and 1.3% had three vessels CAD. In our study, 33.5% patients had single vessel CAD, 13.4% had two vessel CAD and 8.9% had three vessel CAD. In our study, 40.6% patients had normal / mild CAD. The apparent difference between two studies is due to the fact that we have not taken the patients who had less than 50% stenosis as significant CAD but Ali et al. has not mentioned this definition of the CAD in their study. Moreover, the cutoff age was 40 years as compared to 35 years in our study.^18^

Younger patients have totally different disease pattern, so the management should also be different. In only 200(36.2%) patients the disease was severe amongst the younger age group, so one should be very sure about the severity of disease before adopting an invasive strategy, otherwise medical management might be a good option. Clot, spasm, spontaneous coronary artery dissection, myocardial bridging and Takotsubo syndrome may be the cause of acute presentation.^19^ Myocardial infarction in the absence of obstructive CAD (MINOCA) has been well described in the literature and it is also common in young adults.^20^ So, when we take the patient to catheterization lab, without exactly knowing the severity of underlying coronary artery disease the stenting is done leaving behind the lifelong disability of the coronary artery unnecessarily. Sometimes clot/spasm give the impression of severe disease, and PCI or even CABG is done on a non-severe underlying atherosclerotic CAD.

### Limitations of the study:

It is a single center study; the data must be multi center to reflect the true picture of disease pattern in young Pakistani individuals.

## CONCLUSION

Young patients have different coronary artery disease pattern, so the management strategy must be different in this population. One should be very sure about the underlying severity of disease before adopting an invasive strategy. Majority of the young patients have non severe disease. Clot formation is commonly seen in young adults with moderate coronary artery disease.

### Authors’ Contribution:

**AWKF:** Concept, data collection, responsible and accountable for the accuracy and integrity of the work.

**GH:** Helped in manuscript writing and data collection.

**SY:** Manuscript writing.

**WL:** Helped in data analysis.

**SA:** Helped in data collection and preparation of tables.

## References

[1] Brown JC, Gerhardt TE, Kwon E (2022). Risk Factors for Coronary Artery Disease 2021 Jun 5. StatPearls [Internet].

[2] Moshar S, Najibpour R, Mohsenikia M, Bayesh S, Rad KV, Rahimi N (2016). Comparison of angiography findings in iranian patients younger and older than 50 years underwent coronary angiography in Boo-Ali hospital:A cross-sectional study. Thrita.

[3] Abed Y, Jamee A (2015). Characteristics and risk factors attributed to coronary artery disease in women attended health services in Gaza-Palestine observational study. World J Cardiovasc Dis.

[4] Deshmukh PP, Singh MM, Deshpande MA, Rajput AS (2019). Clinical and angiographic profile of very young adults presenting with first acute myocardial infarction:Data from a tertiary care center in Central India. Indian Heart J.

[5] Aggarwal A, Srivastava S, Velmurugan M (2016). Newer perspectives of coronary artery disease in young. World J Cardiol.

[6] Regitz-Zagrosek V, Oertelt-Prigione S, Prescott E, Franconi F, EUGen Med Cardiovascular Clinical Study Group (2016). Gender in cardiovascular diseases:impact on clinical manifestations, management, and outcomes. Eur Heart J.

[7] Anh DT, Minh HV, Binh HA, Bao TQ, Hai NTT, Nam LX (2021). Age Related Differences in Acute Coronary Syndrome:An Observation at a Central Hospital in Vietnam. J Transl Int Med.

[8] Zeitouni M, Clare RM, Chiswell K, Abdulrahim J, Shah N, Pagidipati NP (2020). Risk Factor Burden and Long-Term Prognosis of Patients with Premature Coronary Artery Disease. J Am Heart Assoc.

[9] Khan HU, Khan MU, Noor MM, Hayat U, Alam MA (2014). Coronary artery disease pattern:A comparison among different age groups. J Ayub Med Coll Abbottabad.

[10] Batra MK, Rizvi NH, Sial JA, Saghir T, Karim M (2019). Angiographic characteristics and in hospital outcome of young patients, age up to 40 versus more than 40 years undergoing primary percutaneous coronary intervention. J Pak Med Assoc.

[11] Revaiah PC, Vemuri KS, Vijayvergiya R, Bahl A, Gupta A, Bootla D (2021). Epidemiological and clinical profile, management and outcomes of young patients (≤40 years) with acute coronary syndrome:A single tertiary care center study. Indian Heart J.

[12] Ezhumalai B, Jayaraman B (2014). Angiographic prevalence and pattern of coronary artery disease in women. Indian Heart J.

[13] Mohammad AM, Jehangeer HI, Shaikhow SK (2015). Prevalence and risk factors of premature coronary artery disease in patients undergoing coronary angiography in Kurdistan, Iraq. BMC Cardiovasc Disord.

[14] Maroszyńska-DmochEM, Wożakowska-Kapłon B (2016). Clinical and angiographic characteristicsof coronary artery disease in young adults:A single center study. Kardiol Pol.

[15] Prajapati J, Joshi H, Sahoo S, Virpariya K, Parmar M, Shah K (2015). AGE-Related Differences of Novel Atherosclerotic Risk Factors and Angiographic Profile Among Gujarati Acute Coronary Syndrome Patients. J Clin Diagn Res.

[16] Mahjoob MP, Sadeghi S, Khanaman HF, Naderian M, Khaheshi I (2018). Comparison of coronary risk factors and angiographic findings in younger and older patients with significant coronary artery disease. Rom J Intern Med.

[17] Yang J, Biery DW, Singh A, Divakaran S, DeFilippis EM, Wu WY (2020). Risk Factors and Outcomes of Very Young Adults Who Experience Myocardial Infarction:The Partners YOUNG-MI Registry. Am J Med.

[18] Ali M, Butt UM, Khan RSAT, Qureshi MA, Akram Z (2021). Coronary artery disease in young Pakistanis:risk factors and pattern of disease. Ann King Edward Med Uni.

[19] Waterbury TM, Tarantini G, Vogel B, Mehran R, Gersh BJ, Gulati R (2020). Non-atherosclerotic causes of acute coronary syndromes. Nat Rev Cardiol.

[20] Tamis-Holland JE, Jneid H, Reynolds HR, Agewall S, Brilakis ES, Brown TM (2019). Contemporary Diagnosis and Management of Patients With Myocardial Infarction in the Absence of Obstructive Coronary Artery Disease:A Scientific Statement From the American Heart Association. Circulation.

